# Predominant Founder Effect among Recurrent Pathogenic Variants for an X-Linked Disorder

**DOI:** 10.3390/genes13040675

**Published:** 2022-04-12

**Authors:** Chelsea Bender, Elizabeth Geena Woo, Bin Guan, Ehsan Ullah, Eric Feng, Amy Turriff, Santa J. Tumminia, Paul A. Sieving, Catherine A. Cukras, Robert B. Hufnagel

**Affiliations:** 1 National Eye Institute, National Institutes of Health, Bethesda, MD 20892, USA; chelsea.bender@nih.gov (C.B.); wooeg@uchicago.edu (E.G.W.); bin.guan@nih.gov (B.G.); ehsan.ullah@nih.gov (E.U.); efeng337@gmail.com (E.F.); amy.turriff@nih.gov (A.T.); tumminias@nei.nih.gov (S.J.T.); paulsieving@gmail.com (P.A.S.); cukrasc@nei.nih.gov (C.A.C.); 2 UC Davis Medical Center, Ophthalmology & Vision Sciences, University of California, Davis, CA 95817, USA

**Keywords:** X-linked disorder, X-linked retinoschisis (XLRS), *RS1*, variant classification, ACMG/AMP variant interpretation guideline, founder effect, haplotype analysis

## Abstract

For disorders with X-linked inheritance, variants may be transmitted through multiple generations of carrier females before an affected male is ascertained. Pathogenic *RS1* variants exclusively cause X-linked retinoschisis (XLRS). While *RS1* is constrained to variation, recurrent variants are frequently observed in unrelated probands. Here, we investigate recurrent pathogenic variants to determine the relative burden of mutational hotspot and founder allele events to this phenomenon. A cohort *RS1* variant analysis and standardized classification, including variant enrichment in the XLRS cohort and in *RS1* functional domains, were performed on 332 unrelated XLRS probands. A total of 108 unique *RS1* variants were identified. A subset of 19 recurrently observed *RS1* variants were evaluated in 190 probands by a haplotype analysis, using microsatellite and single nucleotide polymorphisms. Fourteen variants had at least two probands with common variant-specific haplotypes over ~1.95 centimorgans (cM) flanking *RS1*. Overall, 99/190 of reportedly unrelated probands had 25 distinct shared haplotypes. Examination of this XLRS cohort for common *RS1* haplotypes indicates that the founder effect plays a significant role in this disorder, including variants in mutational hotspots. This improves the accuracy of clinical variant classification and may be generalizable to other X-linked disorders.

## 1. Introduction

Disorders of X-linked inheritance represent a significant genetic disease burden. Pathogenic variants on the X chromosome are currently known to cause over 500 X-linked diseases that mainly affect the brain, bone, blood, ears, heart, liver, kidney, retina, skin, and teeth [1]. For X-linked disorders, females may be unaffected carriers of X-linked pathogenic variants, with either no disease or a lesser form of the disease than observed in hemizygous males [2]. Therefore, a variant may be passed through multiple generations of female carriers without ascertaining an affected male. In those cases, an analysis for co-segregation by chance may be limited. As such, proper variant classification within X-linked disorders can be particularly challenging for those disorders where carrier females do not express the phenotype.

Recurrently observed variants in X-linked disorders may represent independent mutation events or a founder allele passed through multiple obligate carrier females [3]. This can be discerned through a haplotype analysis of microsatellite markers and other variations. However, large patient cohorts with an ascertained disease-relevant genetic variation are required to discern the mechanism of each genetic event to support proper variant classification [4].

X-linked juvenile retinoschisis (XLRS, OMIM # 312700) is an X-linked monogenic disease, and it exhibits complete penetrance in males [5]. The phenotype, consisting of central and peripheral retinoschisis with characteristic electronegative responses on electroretinographic testing, is pathognomonic, and >95% of males with XLRS have a pathogenic or likely pathogenic variant in the *RS1* gene (OMIM 300839, Xp22.13). Retinoschisin, encoded by *RS1*, is localized to the neuroretina and critical for the maintenance of photoreceptor-bipolar connections [6]. Heterozygous females generally do not express features of the disease, though minor phenotypes have been reported [7]. The prevalence of XLRS is estimated to be between 1 in 5000 to 1 in 25,000 males [8].

Accurate clinical *RS1* variant classification is critical, as gene therapy clinical trials are ongoing [9]. Pathogenic *RS1* variants include missense, frameshift, nonsense, intronic, and exonic deletions, and many are recurrent. This raises two possibilities: (i) these specific nucleotide sites are prone to genetic alteration or (ii) these represent founder alleles passed silently through female lineages for many generations.

In this study, we assessed a large cohort of unrelated probands with XLRS and associated *RS1* variants to determine the burden of the cryptic founder variation, finding sufficient evidence to apply hotspot, enrichment, and co-segregation criteria for many *RS1* variants.

## 2. Materials and Methods

### 2.1. Consent, Sample Collection, and Testing

Variants from a cohort of 332 XLRS probands, which included patients tested at the National Eye Institute (NEI) Ophthalmic Genetics Clinic at the National Institutes of Health Clinical Center and participants in eyeGENE^®^ (EYEGENE Inc., Seoul, Korea), were analyzed. eyeGENE^®^ is an international registry and DNA repository for patients with inherited ophthalmic diseases and their family members [10,11,12,13]. These studies (NCT02471287 and NCT00378742) were approved by the National Institutes of Health Institutional Review Board and adhered to the tenants of the Declaration of Helsinki. Informed consent was obtained from all participating subjects. Genomic DNA was extracted from peripheral blood. The six exons in the *RS1* (NM_000330.4) coding sequence and flanking intronic regions were amplified by polymerase chain reaction (PCR) (Supplementary Table S1) and sequenced by Sanger sequencing. Additional family members were also tested when available.

### 2.2. Variant Classification

**Figure 1:**
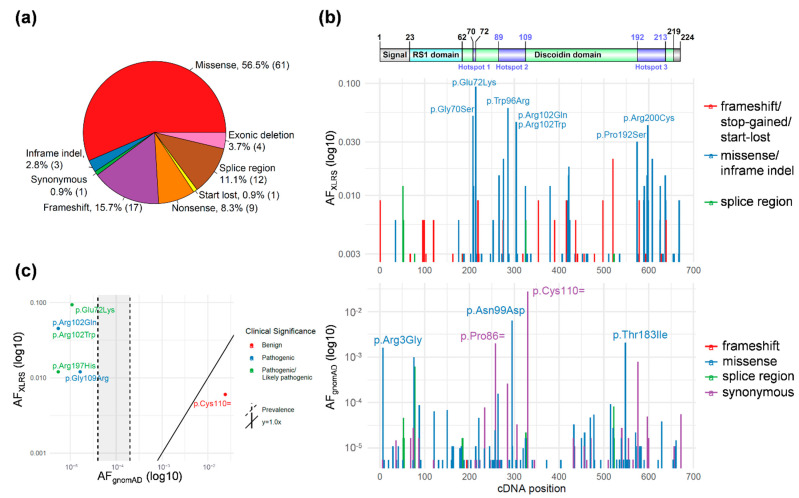
*RS1* variant types and localizations in the XLRS cohort and gnomAD. (**a**) Variant type distribution in the XLRS cohort. (**b**) RS1 protein domains (Top) and variant allele frequencies (AF) along the cDNA position in the XLRS cohort (middle) and gnomAD (bottom). The splice region variants in the XLRS cohort include intronic variants reported in clinical reports. The splice region variants in the gnomAD dataset include intronic variants in ±3–8 nucleotides from the exon–intron junctions. The gnomAD v2.1.1 dataset does not contain any variants in the ±2 canonical splicing sites. Variants denoted are those with AF > 0.03 in XLRS or >0.001 in gnomAD. (**c**) Allele frequencies for variants present in both XLRS cohort and gnomAD. Shaded: the XLRS prevalence of 1 in 5000 to 1 in 25,000 males.

General population *RS1* variant allele frequency data were downloaded from The Genome Aggregation Database (gnomAD v2.1.1) [14]. The Ensembl Variant Effect Predictor (VEP) for GRCh37 was used to annotate additional information, such as the chromosome coordinates, consequence, and HGVS protein sequence name [15]. Variant allele frequencies from gnomAD were compared to allele frequencies in the *RS1* patient cohort and visualized using the ggplot2 package in R version 4.0.2 (Figure 1c). Fisher’s exact test for independence was performed for all *RS1* variants in R. Variant classification was manually assigned according to American College of Medical Genetics and Genomics (ACMG)/Association for Molecular Pathology (AMP) guidelines [16], excluding large exonic deletions (Supplementary Table S2). In-silico pathogenicity predictions were obtained from Varsome [17,18,19,20]. The Human Gene Mutation Database (HGMD) [21] was used to find literature with previously published functional secretion data for variants, for which we assigned a downgraded PS3 (well-established functional studies providing strong support of a pathogenic effect) as moderate evidence for pathogenicity, as this is a research test. ClinVar [22], a submission-based public archive that aggregates information about genomic variations, was accessed (4 January 2022) for existing variant interpretations. Supplementary Figure S1 indicates the overlap between the XLRS cohort, HGMD, and ClinVar. Pedigrees were examined for variant co-segregation evidence [23].

### 2.3. Minigene Assay

The plasmid RHCglo used for the minigene assay was a gift from Thomas Cooper (Addgene plasmid #80169; http://n2t.net/addgene:80169, accessed on 5 January 2022; RRID:Addgene_80169) [24]. A DNA fragment including the *RS1* exon 2 plus 337 bp upstream and 373 bp downstream was synthesized according to the human genome GRCh37 and cloned into RHCglo to make the wildtype RHCglo-RS1-E2 vector, which was then used as a template to make the c.53-34A>G mutant vector by mutagenesis (LifeSct, Rockville, MD, USA). The wildtype and mutant RHCglo plasmids were transfected into the 293FT cells using the Lipofectamine 2000 reagent (ThermoFisher, Waltham, MA, USA) using a suspension transfection method as described previously [25]. RNA was prepared from cells harvested one day post transfection (RNeasy mini kit, Qiagen, Hilden, Germany) and used for cDNA synthesis (iScript cDNA synthesis kit, Bio-Rad, Hercules, CA, USA). M13-tagged PCR primers located in exons in the RHCglo vector were used for PCR amplification of the cDNA, RSV5U_M13F (GTAAAACGACGGCCAGTCATTCACCACATTGGTGTGC), and RTRHC_M13R (CAGGAAACAGCTATGACCGCTTTGCAGCAACAGTAACCAG). The PCR products were then subjected to Sanger sequencing using the BigDye Direct Cycle sequencing kit, followed by analysis using a SeqStudio genetic analyzer (ThermoFisher, Waltham, MA, USA).

### 2.4. Haplotype Analysis

A haplotype analysis was performed on 190/332 probands for 19 of the most recurrent variants, where one variant was observed in at least four probands with DNA available. Haplotypes were determined by analyzing a total of seven markers, three short tandem repeat (STR) markers (~34.75–36.51 cM, chrX:17741821-19054560 GRCh37) and four single nucleotide polymorphism (SNP) markers (~34.56–36.25 cM, chrX: 17568516-18876226 GRCh37), surrounding the *RS1* gene (~1.95 cM, 34.56–36.51 cM, chrX: 17568516-19054560 GRCh37) on the X chromosome (Supplementary Table S3). Highly polymorphic STR markers were evaluated and selected by heterozygosity and proximity to the *RS1* gene from the Rutgers Combined Linkage-Physical Map v3 map [26]. Primer sequences were obtained from UCSC genome browser [27] (https://genome.ucsc.edu/, accessed on 26 March 2018). Primer pairs were designed with M13 tails on the forward primers and combined with M13 tagged 5′ 6-fluorescein amidite (FAM), as described previously [28]. A PCR was performed using proband DNA, OneTaq^®^ Hot Start 2X Master Mix with Standard Buffer (New England Biolabs, Ipswich, MA, USA) and the labeled primers. PCR products were analyzed by capillary electrophoresis using a 3500 Genetic Analyzer (Applied Biosystems, Waltham, MA, USA), marker alleles were called using the GeneMapper ™ software (Applied Biosystems, Waltham, MA, USA), and alleles were determined based on fragment sizes. SNP markers with allele frequencies ranging from ~19% to ~68% were selected from gnomAD v2.1.1 based on the allele frequency in European and Hispanic populations and proximity to the *RS1* gene. Primer sequences were designed using Primer3 (https://bioinfo.ut.ee/primer3-0.4.0/, accessed on 18 November 2018) and engineered with M13 tails. PCR and Sanger sequencing were performed following the BigDye Direct Cycle Sequencing protocol (Applied Biosystems, Waltham, MA, USA). Sequencing products were processed by capillary electrophoresis using a SeqStudio Genetic Analyzer (Applied Biosystems, Waltham, MA, USA). Sequencing data were analyzed using a Mutation Surveyor^®^ (SoftGenetics, State College, PA, USA), and letter-based alleles were assigned to the observed nucleotide at the SNP.

## 3. Results

### 3.1. RS1 Gene-Level Variant Characterization

A total of 108 unique *RS1* variants, including four large exonic deletions, were identified in a cohort of 332 probands with XLRS (Supplementary Table S2). Missense variants accounted for the majority of variants (61/108, 56.5%), followed by nonsense, frameshift, or start loss (27/108, 25%) and splice-region (12/108, 11.1%) variants (Figure 1a). The missense variant c.214G>A p.(Glu72Lys) was found most frequently amongst patients (31/332, 9.3%), followed by missense variants c.286T>C p.(Trp96Arg) (20/332, 6.0%), c.208G>A p.(Gly70Ser) (17/332, 5.1%), c.305G>A p.(Arg102Gln) (15/332, 4.5%), and c.304C>T p.(Arg102Trp) (15/332, 4.5%).

To assess variant pathogenicity in the *RS1* gene, allele frequencies of variants in control and disease populations were compared. The gnomAD data was used as a general population control cohort for the comparison and analysis of the 108 *RS1* variants among 332 XLRS probands. Variant allele frequencies in the XLRS cohort and gnomAD were plotted against the cDNA position to compare the relative allele frequency and location within the cDNA and protein position (Figure 1b). The majority of missense or in-frame indels were found in the *RS1* Discoidin domain in the XLRS cohort. Intriguingly, there were three peaks with allele frequencies above 3% in the patient cohort. We attempted to define potential mutation hotspots involving these residues by extending to neighboring residues and merging into one hotspot if the AF_XLRS_ was above 1% in 10-a.a. sliding windows. Using this strategy, we found three significant hotspots involving a.a. 70–72, a.a. 89–109, and a.a. 192–213 (Supplementary Table S4). The missense or in-frame indel variants in these three hot spots represented 58.4% of the families in the cohort. Interestingly, these hotspots co-localize with intermolecular interface in the *RS1* octamer [29], providing strong genetic evidence for the important functions of the intact *RS1* octamer in normal retinal physiology.

### 3.2. RS1 Population-Level Variant Analysis

Next, we established the variant’s association with disease status by analyzing the variant frequency in the disease cohort compared to the general population. Allele frequencies from both datasets (XLRS proband cohort and gnomAD) were visualized to predict the enrichment of variants in the disease population compared to the control (Figure 1b). Notably, the vast majority of variants in the XLRS cohort were absent from gnomAD, even as heterozygous variants in female samples. Among the 108 *RS1* variants identified in the patient cohort, only six variants, including five missense variants and one synonymous, were also found in the gnomAD general population (Figure 1c). Variants lying above the diagonal y = 1.0x line and to the left of the prevalence line in Figure 1c demonstrated enrichment in the disease population compared to the control population, suggesting pathogenicity. Conversely, variants below the line were found at higher frequencies in the control population, indicating they may be benign. Two were classified as pathogenic in ClinVar (c.325G>C p.(Gly109Arg) and c.305G>A p.(Arg102Gln)) and three as pathogenic or likely pathogenic (c.214G>A p.(Glu72Lys), c.304C>T p.(Arg102Trp), and c.590G>A p.(Arg197His)). One out of the six variants in both the patient cohort and gnomAD was found more frequently in the general population, which was synonymous variant c.330T>C p.(Cys110=). This variant was annotated as benign in ClinVar.

**Figure 2:**
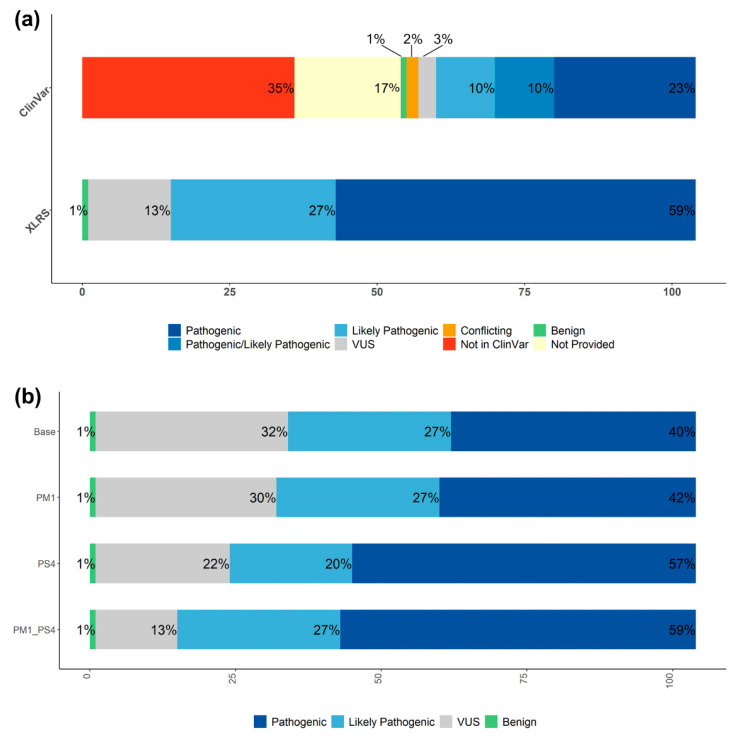
Variant analysis using the ACMG/AMP classification system, including 104 SNVs and small Indels. (**a**) Variant interpretation in the current study as compared to ClinVar interpretations. (**b**) Variant interpretation with and without application of PM1 and PS4.

A Bonferroni-corrected *p*-value of 0.00046 (0.05/108 variants) from Fisher’s exact test was used as the threshold to determine enrichment of a variant in the *RS1* cohort as compared to gnomAD. This analysis identified 49 significantly enriched *RS1* variants, including the five missense variants present in gnomAD. According to the ACMG criterion for variant interpretation PS4, a significant increase in the prevalence of a variant in affected individuals compared with the prevalence in controls is considered strong evidence for pathogenicity [16]. In general, a variant absent in gnomAD and found only in one proband in the *RS1* cohort had a *p*-value of 0.00181; thus, PS4 was not applied. In contrast, a variant absent in gnomAD and recurrent in the *RS1* cohort had a *p*-value less than 0.00046, so PS4 was applied for classification. The application of PS4 allowed for pathogenicity classification of many previously unclassified variants (Figure 2).

Following the variant:disease enrichment analysis, we assessed the co-segregation of a variant with a phenotype or a lack of segregation without a phenotype as additional cohort-level pathogenicity evidence. In the XLRS patient cohort, 49/108 (45.4%) variants were present in at least one family in which more than one individual was tested. Co-segregation analysis provided strong evidence of pathogenicity for eight variants, moderate evidence for one, and supporting evidence for two variants [23] (Supplementary Table S2).

One deep intronic variant, c.53-34A>G, was found in two families affected by XLRS. We applied PS4 (*p*-value = 3.28 × 10^−^^6^, Fisher’s exact test), PP1>PS (upgraded PP1, co-segregated with XLRS in the two families), PM2 (absent in gnomAD), PP3 (predicted to altering splicing with SpliceAI, score 0.65), and PP4 to classify this variant as pathogenic. As this variant alters a predicated lariat branchpoint [30] and lariat branchpoint variants in genetic diseases are relatively rare [31], we performed a minigene assay to test the variant’s effect on splicing in vitro. As shown in Supplementary Figure S2, the variants led to the skipping of exon 2 of *RS1* in the minigene assay, providing moderate evidence (PS3_Moderate) to support this variant as pathogenic.

### 3.3. Many Recurrently Observed RS1 Variants Share Haplotypes

**Figure 3:**
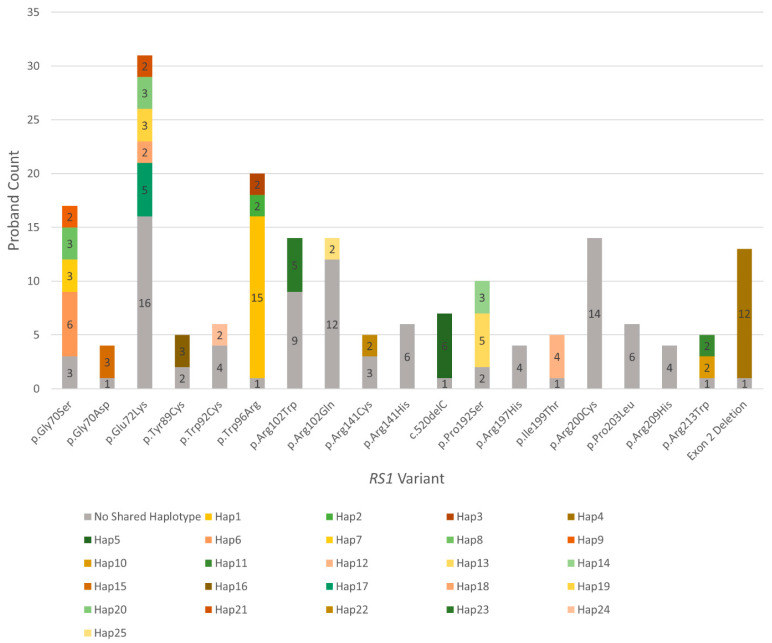
Distribution of distinct shared haplotypes (multi-colored) among 19 recurrently observed *RS1* variants, including probands with no variant-specific shared haplotypes (gray).

Haplotypes can be compared to discern whether frequently observed pathogenic variants among unrelated probands arise from one ancient founder mutation event or sporadically in multiple unrelated events [32]. To investigate the founder effect burden among recurrent *RS1* alleles, we performed a haplotype analysis on reportedly unrelated probands that shared prevalent variants. Fourteen of the 19 recurrent variants tested and 99/190 (52.1%) probands had at least two probands with variant-specific haplotypes that shared all 7 markers (~1.95 cMor 1486 kb) (Figure 3 and Supplementary Table S5); 10/190 (5.3%) probands also had two probands that shared a haplotype but had no shared variant (haplotypes 26–30 in Supplementary Table S5). The most prevalent variant observed in this cohort was c.214G>A (p.Glu72Lys), which has been previously identified as a Finnish founder variant [33]. In this cohort, 15/31 probands with p.Glu72Lys had five distinct haplotypes where at least two probands shared all markers. The second most recurrent variant, c.286T>C (p.Trp96Arg), had 19/20 probands with three distinct haplotypes that shared all markers. Another prevalent variant was c.208G>A (p.Gly70Ser), where 14/17 probands had four distinct haplotypes that shared all markers. A summary of all tested probands and their corresponding haplotypes is listed in Supplementary Table S6.

**Figure 4:**
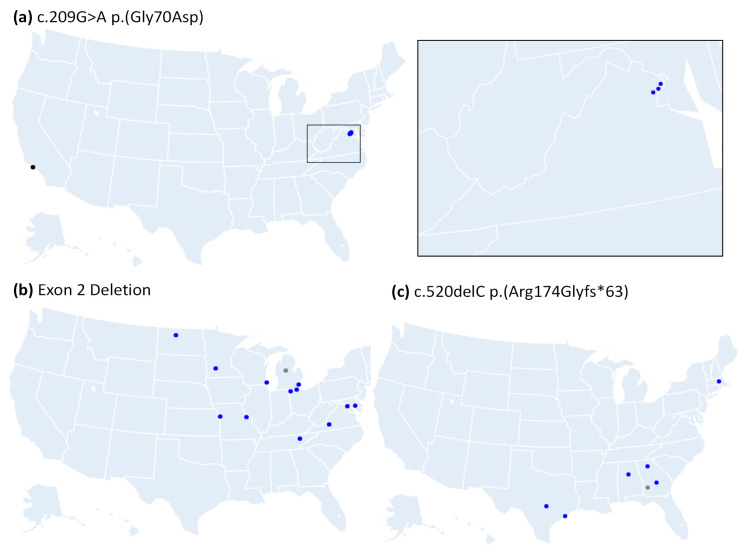
Geographical distribution of three recurrent variants by proband ZIP code. (**a**) Three probands with variant-specific haplotypes (Hap15) tightly cluster (blue inset); one proband with the same *RS1* variant shared 2/7 haplotype markers (gray). (**b**) Twelve probands with variant-specific haplotypes are in blue (Hap4); one proband with the same *RS1* variant shared 6/7 haplotype markers (gray). (**c**) Six probands with variant specific haplotypes are in blue (Hap5), and one proband shared 6/7 haplotype markers (gray).

Furthermore, home ZIP codes of probands with the recurrent variants were mapped, and varying degrees of some haplotype-specific clustering was observed (Figure 4 and Supplementary Figure S3). A novel variant, c.209G>A (p.Gly70Asp), was observed among four unrelated probands, where 3/4 probands shared all markers. The three probands with the shared markers were of Bolivian descent, and their home ZIP codes clustered tightly in Virginia (Figure 4a), while the fourth proband shared 2/7 markers, was of European descent, and had a Californian ZIP code. Haplotype analysis was done for 13 probands with the exon 2 deletion, which showed that 12 shared all markers and that the 13th shared 6/7 markers; the probands with this shared variant loosely cluster in the North-East (Figure 4b). Six of the probands with the c.520delC (p.Arg174Glyfs*63) variant shared all markers, and the seventh shared 6/7; these probands also loosely clustered in the South-East (Figure 4c). Supplemental Figure S3 demonstrates more complex examples of the three most recurrent variants with multiple shared haplotypes.

### 3.4. Systematic RS1 Variant Classification and Interpretation

Following application of population enrichment and familial disease:variant co-segregation evidence, a systematic classification using ACMG/AMP clinical variant interpretation criteria was performed. We were able to apply supporting evidence PP4 to all variants, since *RS1* was the only gene associated with XLRS. Most variants were absent in gnomAD, allowing for the application of PM2. About half of the variants were significantly enriched in the patient cohort compared to the control, allowing for application of PS4, while the other half were present in only a single patient. The synonymous variant found in exon 5, c.330T>C p.(Cys110=), was considered benign as it was found in two probands, in cis with exon 4 variant c.189C>A (p.Cys63Ter) in one patient and with exon 4 variant c.216G>C (p.Glu72Asp) in another. The missense variant, c.295A>G (p.Asn99Asp), in exon 4 was also found in cis with the pathogenic missense variant c.608C>T (p.Pro203Leu).

Excluding large deletions, 54/104 (51.9%) variants from the patient cohort had unavailable existing variant interpretations in ClinVar, and 5/104 (4.8%) were in ClinVar but had conflicting interpretations or were variants of uncertain significance (VUS) (Figure 2a). Of the remaining variants from the patient cohort that did have available interpretations on ClinVar, 24/104 (23%) were classified as pathogenic, 10/104 (9.6%) as pathogenic/likely pathogenic, and 10/104 (9.6%) as likely pathogenic, while 1/104 variant (0.9%) was benign. After manual interpretation of the 104 *RS1* patient cohort variants (excluding the four large deletions), 61/104 (59%) were classified as pathogenic, 28/104 (27%) as likely pathogenic, and 1/104 (1%) as benign, with 14/104 (13%) remaining as VUS (Figure 2b). Twelve of these 14 manually-classified variants were also either unavailable, VUS, or not provided in ClinVar. Of the 59 variants from the patient cohort without interpretation or with conflicting or uncertain interpretation in ClinVar, manual evaluation led to the classification of 31 (30%) as pathogenic and 16 (15%) as likely pathogenic, with 12 (11.5%) remaining as variants of uncertain significance. Thus, this cohort-level approach improves variant classification at the gene-level.

## 4. Discussion

This comprehensive *RS1* variant classification dataset revealed that founder alleles were more common than independent events for a majority of the tested recurrent *RS1* variants. Our analyses greatly expanded the number and depth of clinical interpretation for *RS1* variants associated with XLRS and can be applied to other disease genes. This study demonstrates the benefit of using large patient cohorts to analyze variants at the population-level. Following manual interpretation, 61/104 variants were classified as pathogenic and 28/104 as likely pathogenic, with 14/104 remaining VUS, as opposed to 59/104 variants for whom clinical significance was either absent, not provided, conflicting, or uncertain in ClinVar.

The *RS1* gene encodes a 224 amino acid (a.a.) polypeptide consisting of the N-terminal signal sequence (a.a. 1–23), the *RS1* domain (a.a. 24–62), the Discoidin domain (a.a. 63–219), and a short C-terminal segment (a.a. 220–224). The secreted *RS1* protein forms octamers that play important roles in cell–cell adhesion in the retina [29]. The predicted loss of function variants, including frameshift and nonsense variants, from the patient cohort were distributed throughout cDNA positions, while the missense or in-frame indel variants were mostly present in the Discoidin domain. The *RS1* gene is intolerant to truncating variation, with a pLI score of 0.96 in gnomAD, and it is relatively intolerant to missense variation, with a Z-score of 0.97. The variants from gnomAD were distributed throughout all cDNA positions with a summative allele frequency of 0.012 for all of the missense variants. In this XLRS cohort analysis, we established statistical mutational hotspot support for three regions, a.a. 70–72, 89–109, and 192–213, conferring moderate evidence for variant pathogenicity. However, this could represent either a founder effect or a mutational hotspot. For instance, the c.214G>A p.(Glu72Lys) variant is located in a CpG site on the reverse strand, where a C>T mutation can occur spontaneously [34], and a haplotype analysis showed that ~48% (15/31) of probands tested had five distinct shared profiles.

The vast majority of missense alleles were present in the *RS1* Discoidin domain. One resided in the signal peptide, c.35T>A p.(Leu12His), which was found in two probands and absent in gnomAD. This variant has been previously shown to abolish *RS1* secretion [35]. Another two involved the same residue in the *RS1* domain, c.176G>A p.(Cys59Tyr) and c.176G>C p.(Cys59Ser), which was also found in 2 and 1 probands, respectively, and absent in gnomAD. The p.Cys59 residue is modeled to form an intermolecular disulfide bond with p.Cys223 in another *RS1* subunit in the octamer [29,36].

Founder effects among XLRS variants have been reported previously. The highest carrier frequency of XLRS is in the Finnish population at 14 per 10,000 or 1 in ~700 individuals, where three founder variants, p.Glu72Lys, p.Gly109Arg, and p.Gly74Val, were thought to contribute to the majority of this prevalence [33,37]. Because female XLRS carriers are asymptomatic and the penetrance of *RS1* is nearly complete in males, studying the allele frequency of the *RS1* pathogenic variant in the female population in gnomAD may offer an opportunity to provide a relatively accurate estimate of XLRS prevalence. There are 11 retinoschisis-associated *RS1* variants recorded in the HGMD database that are also found in gnomAD (Supplementary Table S7), including five pathogenic or likely-pathogenic variants present in our XLRS cohort, one uncertain, and another five classified as pathogenic or likely-pathogenic according to the ACMG/AMP classification criteria. The sum allele frequency of these 10 pathogenic or likely-pathogenic variants in gnomAD females is 0.000167, indicating an XLRS prevalence of 1 in 5982 males (Supplementary Table S8). Considering populations separately, the XLRS prevalence is highest in Finnish populations (1:1746), followed by South Asian (1:7541), East Asian (1:9320), African (1:10,050), and non-Finnish European (1:25,075) populations.

These estimates are at the lower end of true prevalence, considering there could be pathogenic variants that were not curated in HGMD. Furthermore, the female populations in gnomAD might not be large enough to cover all variant carriers and to provide an accurate estimate. Nevertheless, our analysis refined the disease prevalence of XLRS in diverse populations, which was largely in-line with previous estimates based on epidemiology case studies.

The pathogenicity assessment of these variants has exciting potential for use in clinical settings, with applications in precision medicine and the diagnosis of genetic disorders. Variant interpretation is a time-limiting step in the process, however, and it requires support from multiple types of evidence. In 2015, ACMG and AMP published standards and guidelines for variant classification [16]. Population data is an important category of evidence in these guidelines for pathogenicity assessment. The analysis of allele frequencies in reference population databases has proven to be a useful tool in the assignment of pathogenicity to variants. Furthermore, the use of these reference databases for the comparison of disease and control populations can be informative in predicting pathogenicity for novel variants detected in patients.

The comparison of variant allele frequency with general population data from gnomAD allowed us to identify variants enriched in individuals with the disease as compared to individuals not selected for a particular disease, with evidence strongly suggesting pathogenicity. This study also highlights the value of a pedigree analysis for co-segregation of a variant with a disease. The predicted loss-of-function variants in *RS1* can all be classified as pathogenic because of the application of PVS1, PM2, and PP4 criteria. While co-segregation and population data have a potential to provide strong evidence of variant pathogenicity, the lack of evidence from singletons led to the classification as variants of uncertain significance. In this case, additional evidence, such as functional studies, are necessary for their pathogenicity assessment. The testing of additional family members could also provide supporting or moderate-level co-segregation evidence, possibly allowing for the classification of current VUS or increasing the confidence of variants assigned as “likely pathogenic” to “pathogenic”.

Observation of the founder phenomenon is variable across X-linked disorders and retinopathies. In X-linked Retinitis Pigmentosa-3 (RP3, OMIM # 300029), most variants arise independently [38]. No highly recurrent variants have been reported to date for Mucopolysaccharidosis II [39] (MPS2 or Hunter Syndrome, OMIM # 309900) or Duchenne Muscular Dystrophy (DMD, OMIM # 310200), and only one founder variant has been reported [40] in Becker Muscular Dystrophy (BMD, OMIM # 300376), with the less severe version of DMD involved with the same gene. DMD also tends to have a higher incidence of de novo variants [41]. However, multiple founder variants have been identified in X-linked disorders, such as Congenital Stationary Night Blindness [42] (CSNB, OMIM # 310500) and Fabry Disease [43] (FD, OMIM # 301500), though it is noted that most variants also arise independently.

Multiple recurring *RS1* variant-specific haplotypes were observed among unrelated probands, suggesting that a significant number of these evaluated variants are ancient founder variants. While de novo *RS1* variants have been reported [34,44], they are relatively rare in the XLRS population [34]. Additionally, other XLRS studies have identified other founder variants by haplotype analysis [33,34]. The identification of founder variants in unrelated probands can influence ACMG variant classification by the potential overlap of PP1_Strong and PS4 criteria, as a familial segregation analysis only supports linkage evidence rather than the pathogenicity evidence that the enriched diseased population presents. Determining whether a variant arises due to the founder effect, or a de novo hot spot is critical, as it has implications for female carriers and their families.

## 5. Conclusions

The comparison of variants in large patient and control cohorts provides valuable population-level evidence for variant pathogenicity classification. A pedigree analysis for co-segregation evidence also serves as a helpful tool for classification, with the potential to provide strong evidence, particularly for cryptic founder alleles for X-linked disorders. Additional evidence, such as functional studies, is still needed for further classification of current variants of uncertain significance. This method of analysis could be similarly applied to variants in other disease-associated genes.

## Data Availability

Variant information will be deposited to ClinVar. All other materials will be provided upon reasonable request.

## Supplementary Materials

The following supporting information can be downloaded at https://www.mdpi.com/article/10.3390/genes13040675/s1. Figure S1: Venn diagram depiction of unique *RS1* variants in the study cohort (blue) and variants reported in HGMD (white) and ClinVar (gray); Figure S2: The deep intronic variant c.53-34A>G causes exon skipping; Figure S3: Geographical distribution of three recurrent variants by proband ZIP code; Table S1: List of primer sequences for amplification of the six exons in the *RS1* coding sequence and flanking intronic regions; Table S2: List of unique *RS1* variants in this study with their clinical classification and annotation evidence; Table S3: STR and SNP markers used for *RS1* haplotype analysis; Table S4: Hotspot determination of the pathogenic *RS1* variation comparing segmental variation rates in the XLRS cohort and general population; Table S5: Haplotypes organized by recurrently observed *RS1* pathogenic variants; Table S6: List of 190 XLRS probands with recurrent *RS1* pathogenic variants and haplotype information; Table S7: Gender-specific gnomAD allele frequency of retinoschisis-associated *RS1* variants in HGMD (HGMD professional version 2021.4); Table S8: The XLRS prevalence estimate among populations using the gnomAD dataset.
