# Supplementary figures and images for: Predominant Founder Effect among Recurrent Pathogenic Variants for an X-Linked Disorder
Source: Genes (Basel). 2022 Apr 12;13(4):675. doi: 10.3390/genes13040675 (PMC9029724; doi:10.3390/genes13040675)

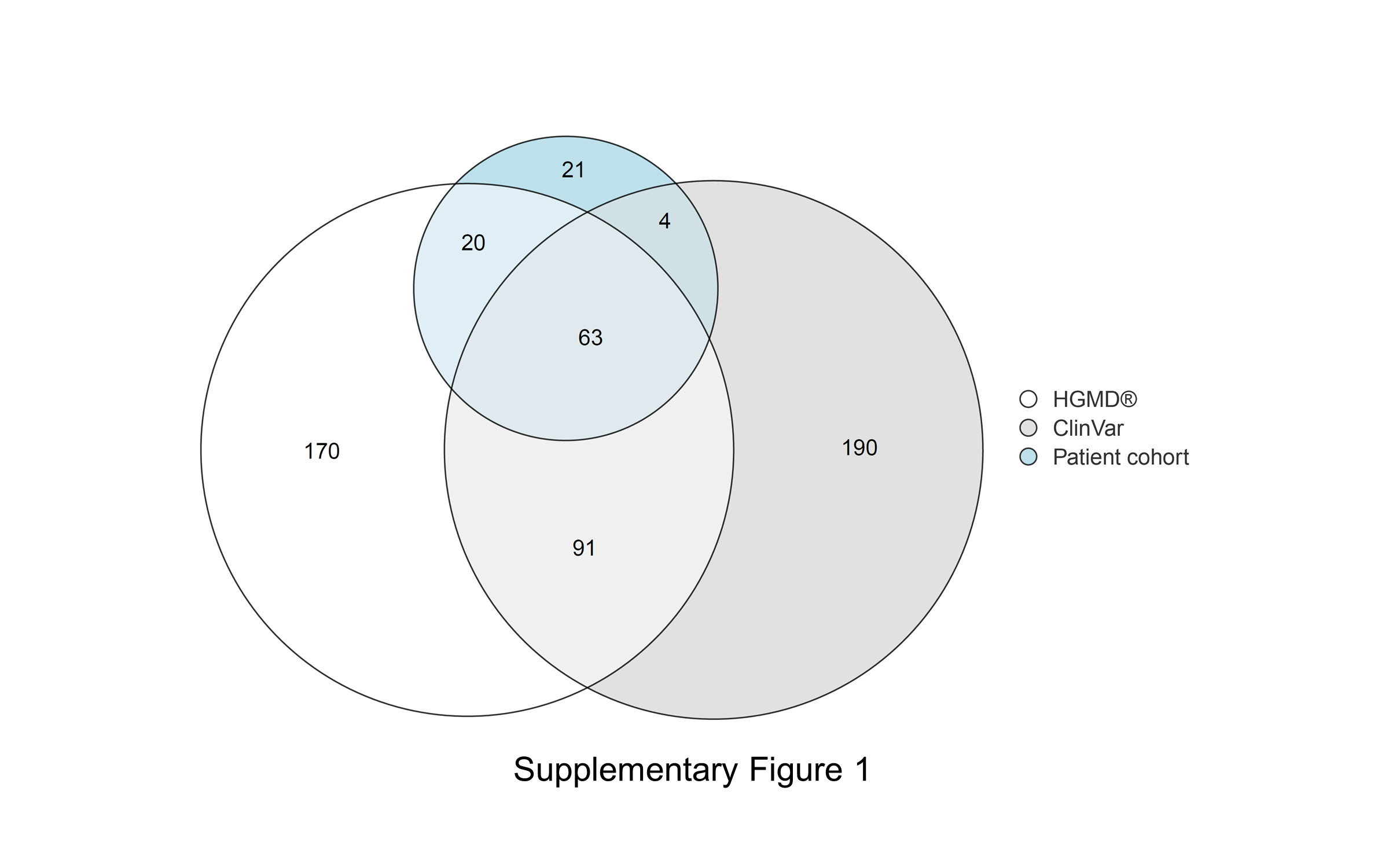

Supplement: Supplementary file 1 [file genes-13-00675-s001.zip › Supplementary-Figure S1.tif]

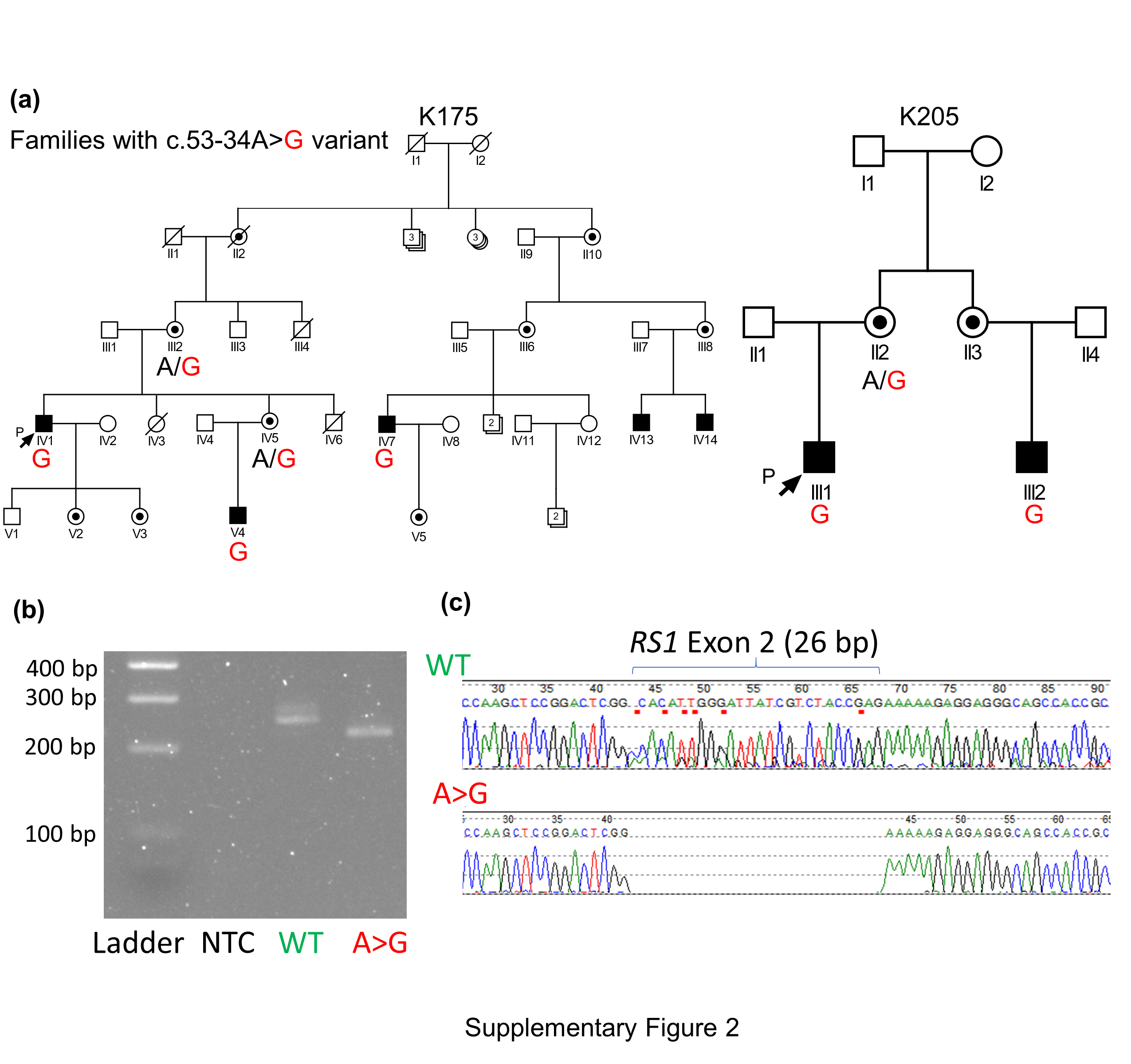

Supplement: Supplementary file 1 [file genes-13-00675-s001.zip › Supplementary-Figure S2.tif]

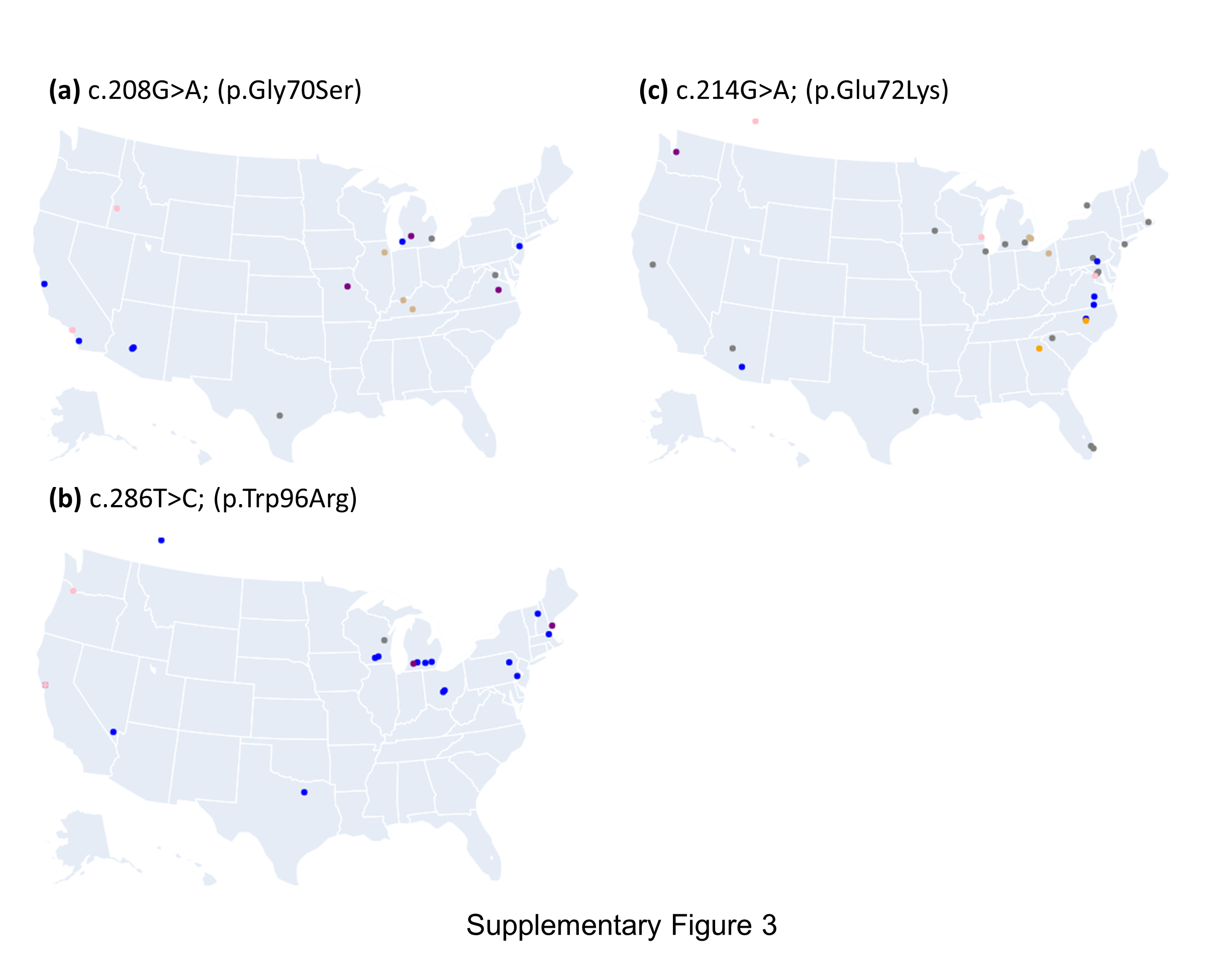

Supplement: Supplementary file 1 [file genes-13-00675-s001.zip › Supplementary-Figure S3.tif]
